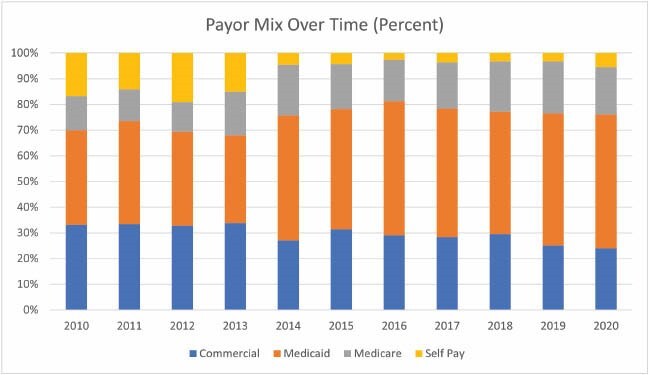# 95 Shifting Payor Mix May Challenge Financial Sustainability of Burn Centers

**DOI:** 10.1093/jbcr/irad045.068

**Published:** 2023-05-15

**Authors:** Jamie Oh, Clifford Sheckter, Barclay Stewart, Virginia Wang

**Affiliations:** University of Washington, Seattle, Washington; Stanford University, Palo Alto, California; University of Washington, Seattle, Washington; University of Washington, Seattle, Washington; University of Washington, Seattle, Washington; Stanford University, Palo Alto, California; University of Washington, Seattle, Washington; University of Washington, Seattle, Washington; University of Washington, Seattle, Washington; Stanford University, Palo Alto, California; University of Washington, Seattle, Washington; University of Washington, Seattle, Washington; University of Washington, Seattle, Washington; Stanford University, Palo Alto, California; University of Washington, Seattle, Washington; University of Washington, Seattle, Washington

## Abstract

**Introduction:**

The financial sustainability of regional burn centers is essential to the health of the populations they serve, and to the broader development of burn prevention and care innovations. Additionally, per capita out-of-pocket personal health expenditures (OOP) have risen steadily over the last decade. Lower pre-injury income has been shown to predict poorer post-injury quality of life and higher risk of financial toxicity. Given these multi-level dynamics, we aimed to characterize the changing payor landscape and OOP from 2010-2020 at an American Burn Association-verified center to delineate the financial implications of these changes on the health system and per-patient financial liability.

**Methods:**

We merged burn registry and financial data from 2010 to 2020. Financial variables included payor status, pay-to-charge ratio (% of insurance reimbursement/total hospital charges), financial liability (remainder of charges after insurance reimbursement), and patient payment (dollars). Analyses of trend were performed using Pearson’s chi-squared analysis for counts and ANOVA for means.

**Results:**

The percentage of self-pay patients decreased from 2010 to 2020 from over 13% to under 4% of the total number of patients annually (p< 0.001), corresponding with an increase in the percentage of Medicaid patients from 30% to over 40% of the total number of patients annually (p< 0.001). This change became significantly more pronounced in 2014. Notably, most major provisions of the Affordable Care Act were phased in by January 2014, including state-level Medicaid eligibility expansion. Medicaid consistently displayed a lower pay-to-charge ratio (30-40%) than both commercial insurance (~70%) and Medicare (50-80%) across the decade (p< 0.001). Total financial liability increased across the decade for commercial insurance (p< 0.001), Medicaid (p=0.013), and Medicare (p=0.015). From a patient perspective, the average payment burden did not change significantly across the decade despite rising healthcare costs. The average per-patient owed amount ranged from $0-$1000 for self-pay, $150-$200 for Medicare patients, and $500-$600 for patients with commercial insurance.

**Conclusions:**

Burn centers must remain financially sustainable to continue serving their communities. The shifting landscape of healthcare with a population increasingly reliant on Medicaid impacts a hospital’s financial sustainability. This change demonstrates that minor differences in OOP for the average patient may have large implications for hospital systems given the low pay-to-charge ratio of Medicaid.

**Applicability of Research to Practice:**

This work highlights the changing payor landscape and the impact it may have on burn center financial sustainability and risk of patient financial toxicity, particularly among already vulnerable Medicaid recipients.